# Differences in the response mechanism of cadmium uptake, transfer, and accumulation of different rice varieties after foliar silicon spraying under cadmium-stressed soil

**DOI:** 10.3389/fpls.2022.1064359

**Published:** 2023-01-10

**Authors:** Junyang Zhao, Baoshan Yu, Xueli Wang, Lihong Chen, Kashif Akhtar, Shide Tang, Huaming Lu, Jinhua He, Ronghui Wen, Bing He

**Affiliations:** ^1^ State Key Laboratory for Conservation and Utilization of Subtropical Agro-bioresources/Guangxi Key Laboratory of Agro-Environment and Agric-Products Safety, College of Agriculture, Guangxi University, Nanning, China; ^2^ College of Life Science and Technology, Guangxi University, Nanning, China; ^3^ Soil and Fertilizer Workstation, Department of Agriculture and Rural Affairs of Nanning, Guangxi Zhuang Autonomous Region, Nanning, China

**Keywords:** Cd pollution, foliar Si application, rice yield, Cd accumulation, Cd translocation

## Abstract

Most studies have shown that foliar silicon (Si) spraying can reduce the risk of rice quality safety caused by cadmium (Cd) contamination. However, it has recently been found that different rice varieties have different responses to Si. Therefore, we selected six rice varieties (YHSM, YXY1179, YXYLS, JLK1377, MXZ2, and YLY900) to compare the differences in the effects of leaf spray on Cd accumulation among different varieties. According to the change in Cd content in brown rice after Si application, the six rice varieties were divided into two types: Si-inhibited varieties (JLY1377, MXZ2, LY900, and YXYLS) and Si-stimulated varieties (WY1179 and YHSM). For Si-inhibited varieties, the Cd content of rice was reduced by 13.5%–65.7% after Si application. At the same time, the Cd content of the root, stem, leaf, panicle, and glume decreased to different degrees, the Cd content of the cell wall component increased by 2.2%–37.6%, the extraction state of Cd with strong mobile activity (ethanol-extracted and deionized water-extracted) was changed to the extraction state of Cd with weak mobile activity (acetic acid-extracted and hydrochloric acid-extracted), and the upward transport coefficient of different parts was reduced. For Si-stimulated varieties, Si application increased the Cd content of rice by 15.7%–24.1%. At the same time, the cell soluble component Cd content significantly increased by 68.4%–252.4% and changed the weakly mobile extraction state Cd to the strong mobile extraction state, increasing the upward transport coefficient of different sites. In conclusion, different rice varieties have different responses to Si. Foliar Si spraying inhibits the upward migration of Cd of Si-inhibited varieties, thereby reducing the Cd content of rice, but it has the opposite effect on Si-stimulated varieties. This result reminds us that we need to consider the difference in the effect of varieties in the implementation of foliar Si spraying in remediation of Cd-contaminated paddy fields.

## 1 Introduction

Cadmium (Cd) is a heavy metal element that has a strong biological toxicity. Cd in soil mainly comes from human activities such as industrial production, agricultural irrigation, pesticide production, municipal solid waste, and transportation (Tang et al., 2018). The “Bulletin of National Survey on Soil Pollution Status” reported that the over-standard rate of agricultural land in China reached 16.1%. Heavy metal pollution accounts for about 82.8% of the total pollution, out of which Cd (7%) is higher than the other heavy metals (Ministry of Environmental Protection and Ministry of Land and Resources of the People's Republic of China, 2014). In addition, the high mobility of Cd in the soil-rice system allows it to accumulate in a large amount in the rice and transported to the grain and eventually causing the Cd rice problem. More than 60% of the population in China uses rice as staple food (Cheng et al., 2006). Therefore, the high yield, quality, and safety of rice are of very important strategic significance (Arao and Ishikawa, 2006).

Once Cd enters the soil, it is easily absorbed by crops, causing adverse effects such as slow growth and decreased yield (Nocito et al., 2011; Zeng et al., 2017). The lower concentrations of Cd can promote the growth of rice, but when the Cd content in the environment exceeds a certain threshold, it affects the absorption of water and nutrients by rice and inhibits the growth and development of rice (Rizwan et al., 2016; Chen et al., 2018; Rizwan et al., 2018). Generally manifested as growth retardation, short plants, curled leaves turning yellow and chlorosis, yellow-brown stripes, reduced tillering rate, reduced biomass, and even death, which seriously affects rice yield and quality (Rizwan et al., 2012; Hu et al., 2013; Wang et al., 2014; Shahid et al., 2017a).

The main transport processes of Cd accumulation in rice are as follows: root absorption, root-to-bud translocation caused by xylem flow, redirection transport at the nodes, and remobilization in the leaves (Uraguchi and Fujiwara, 2013). Liu et al. (2007) showed that Cd in rice was to a certain extent controlled by the absorption of Cd by plants and the transport of Cd from the root to the stem and to a greater extent influenced by the transport of Cd from the stem to the grain. Kashiwagi et al. (2009) studied the transport, accumulation, and genetic regulation of Cd in the aboveground part of rice. It was found that the content of Cd in grains was determined by the Cd accumulated in the leaves and stems before the heading stage, while from the nutrition stage to the heading stage of rice, the Cd absorbed by the roots would transfer to the upper ground and accumulate in the leaves and stems. Meanwhile, a large number of studies have shown that due to different genotypes, different rice varieties can absorb, accumulate, and distribute Cd in paddy soils between species (Ekmekci et al., 2008; Xu et al., 2010; Pan et al., 2019). However, the sensitivity and transport ability of hybrid rice to Cd were stronger than those of conventional rice, and the ability of Cd accumulation in late rice grains was stronger than that of early rice (Tang et al., 2018).

The leaf is an important nutrient organ of rice; it not only can produce organic matter (OM) through photosynthesis but also can absorb trace elements on the leaf surface (Perrier et al., 2016). Foliar control agents (FCAs) have a good effect on inhibiting the absorption of heavy metals in crops, improving the tolerance and resistance of crops to heavy metals (Vaculík et al., 2012). In recent years, it has received extensive attention because of its high bioavailability, good application effect, convenient use, and high return rate (Cheng et al., 2006). At present, the main effective components of FCAs are beneficial elements of rice such as Si, selenium, and zinc. Liao et al. (2016) showed that applying Si could significantly reduce the Cd content in brown rice through field studies. Chen et al. (2018) proved that spraying 5–25 mM nano Si could significantly reduce the Cd content in rice grains and cobs by 31.6%~64.9% and 36.1%–60.8%, respectively.

Si is the second most important element in the soil after oxygen and important in the growth and development of plants. Together with nitrogen (N), phosphorus (P), and potassium (K), it is one of the “four elements” necessary for rice. It improves the growth, development, photosynthetic capacity, resistance, and the quality of rice (Wang et al., 2010). Si can activate the antioxidant defense system, reduce oxidative damage, reduce cell membrane permeability and free radical damage to the cell membrane, improve the photosynthetic system, and remove heavy metals in cells, thus reducing the toxicity of heavy metals (Fatemi et al., 2020; Liu et al., 2020; Tripathi et al., 2021). Li et al. (2020) found that foliar Si spraying can reduce the content of Cd in rice stems, improve the photosynthesis of rice leaves, and reduce the transport of Cd from stems to brown rice. Xu et al. (2016) used pot experiments and found that Si application reduced the content of exchangeable Cd that has a strong mobility in rice and reduced the toxic effect of Cd on rice. Guo et al. (2022) found that the concentration of Cd in various cell wall components (pectin, hemicellulose, and residues) of leaves increased by 137%~160% after Si spraying. In conclusion, the decrease of Cd content in rice by Si fertilizer application is mainly related to Si reducing the exchangeable Cd and inhibiting the transport of Cd from the root to the shoot.

Foliar spraying of Si fertilizer is a common technique to inhibit Cd uptake and accumulation in rice. In the process of large-scale implementation, we found that even in the adjacent areas with little difference in soil properties and Cd content. The application of Si fertilizer could not achieve the effect of Cd reduction in all fields of rice; on the contrary, the Cd content of rice in some fields would increase significantly. Therefore, we speculate that it may be related to rice varieties, that is, different rice varieties have different responses to Si. Therefore, we planned to compare the foliar application of Si fertilizer performance on the uptake, translocation, and accumulation of Cd and Si and yield of six different varieties of rice to further explore the response mechanism of different rice varieties to Si and its relationship with Cd accumulation. Once we master the key factors of rice response to Si, it will help to accurately guide the safe application of FCAs in Cd-polluted fields.

## 2 Materials and methods

### 2.1 Experimental site

The experiment was carried out in the glass greenhouse of Guangxi University from July 2020 to November 2020. The temperature and humidity of the greenhouse are consistent with the local natural environment with an average annual temperature of about 21.6°C.

### 2.2 Experiment design and treatments

Six different rice varieties were selected. All six varieties were indica rice, of which two varieties [Yuehesimiao (YHSM) and Meixiangzhan (MXZ2)] were conventional rice and the other four varieties [Yexiangyoulisi (YXYLS), Jingliangyou1377 (JLY1377), Wuyou1179 (WY1179), and Yliangyou900 (YLY900)] were hybrid rice.

The soil used for the pot experiment was obtained from the experimental field of Guangxi University in Nanning, Guangxi Zhuang Autonomous Region, China. The soil was sun dried, debris was removed, and the soil was passed through a 20-mm nylon sieve. Cadmium chloride (CdCl_2_•2H_2_O) is used as the source of Cd in the soil. Dissolve the Cd in water, spray it evenly on the soil, and mix it thoroughly. The soil was incubated for 1 month by repeated rehydration. The final total Cd content of the soil is 1.20 mg/kg (the pollution level is in the second category of agricultural land in China, belonging to the medium and high pollution level). The basic physical and chemical properties of soil are shown in **Table 1**.

**Table 1:** Basic physical and chemical property in mg/kg of soil.

pH	Organic matter	Hydrolyzable N	Available P	Available K	Total Cd	Available Cd	Available Si
6.6	18.93	182	31.72	21.36	1.2	0.51	245

The experiment was carried out in a black cylindrical plastic bucket (35 cm × 25 cm × 35 cm). Each bucket was loaded with 5.5 kg of soil. Base fertilizer (urea: 200 mg kg^-1^; potassium dihydrogen phosphate: 130 mg kg^-1^; potassium chloride: 200 mg kg^-1^) was applied to the soil and thoroughly mixed. Three-leaf and one-hearted rice seedlings with similar morphology and good growth are selected and moved into pots with eight plants per pot and three replicates for each treatment. Conventional management techniques were used for top dressing and pest control. Foliar sprays were carried out twice at the tillering stage and filling stage of rice with the FCA. The main component of the FCA is nano-silica, which is provided by Foshan Tieren Environmental Protection Technology Co., Ltd. The concentration is the manufacturer’s recommended dosage (1.7 g L^-1^). Use a handheld watering to spray on the rice surface until the rice leaves are evenly moistened. The average amount of water sprayed per pot is 600 ml. The control group was sprayed with deionized water.

### 2.3 Sample collection and preparation

The parts of the rice samples were collected during the two growing periods (filling and maturity). The roots were soaked in 5 mmol/L EDTA-Na_2_ solution for 20 min to remove the Cd on the root surface and then repeatedly rinsed with deionized water until cleaned. The aboveground parts were rinsed with tap water and then washed twice with deionized water. Some fresh leaves were frozen in liquid nitrogen and stored at -80°C to analyze the subcellular distribution and the form classification of Cd. The other samples were placed in an oven and quenched at 105°C for 30 min and then dried at 70°C to constant weight. After drying, the rice parts (roots, stems, leaves, panicles, and glumes) were separated and pulverized to determine the content of Cd and Si.

### 2.4 Measured index and methods

#### 2.4.1 Determination of the basic physicochemical properties of the soil

The physical and chemical properties of the soil were determined according to the method described in the Analysis Methods of Soil Agricultural Chemistry (Bao, 2000). The pH was measured by the potentiometric method with soil/liquid ratio of 1.0:2.5. The OM content was measured by the potassium dichromate external heating method. Alkaline N was determined by the alkaline diffusion method. Available P was determined by the 0.05 mol/L HCl-0.025 mol/L (1/2H_2_SO_4_) method. Available K was leached by 1 mol/L NH_4_OAC and determined by flame photometric method. Extract available Si from soil with 0.25 M citric acid and analyze it with Si molybdenum blue spectrophotometry. The total Cd content in soil was determined by 2:2:1 HNO_3_:HClO_4_:HF (v:v:v) digestion. The effective state Cd content was extracted by Diethylenetriamine Pentaacetate (DTPA) solution, and the Cd contents of the digestion solution and extract solution were determined using an atomic absorption spectrophotometer (PinAAcle 900T, PerkinElmer, USA).

#### 2.4.2 Determination of the cadmium content in rice

Cd content determination refers to Pan Yao’s method (Hu et al., 2016). The brown rice dry sample passing the 100-mesh sieve was digested with high-grade pure concentrated nitric acid by microwave digestion (microwave digestion instrument, CEM Company, MARS). The Cd content was measured with graphite furnace atomic absorption spectrophotometer (PinAAcle 900T, Platinum Elmer, USA), and the quality was controlled with reference materials.

#### 2.4.3 Determination of the subcellular distribution of cadmium in rice leaves

Use differential centrifugation to separate leaf subcellular components (Liu et al., 2018). Weigh 0.5 g of the sample, grind it in the extract (250 mmol/L sucrose, 1 mmol/L dithioerythritol, 50 mmol/L Tris-HCl) at 4°C, collect the homogenate, and centrifuge (3,000 rpm) for 15 min; the sediment is the cell wall part, and the supernatant is the protoplast component. The supernatant (2,000 rpm) was centrifuged for 15 min to precipitate organelles. The supernatant was the soluble part containing vacuole contents, ribose, protein, etc. All operations were carried out at 4°C. Evaporate the extracted parts to dryness and determine the Cd content according to the method in *Determination of the Cadmium Content in Rice*.

#### 2.4.4 Determination of the chemical form of cadmium in rice leaves

The chemical reagent stepwise extraction method was used to extract different forms of Cd in sequence (Shahid et al., 2017b). The five extracts are as follows: 1) ethanol extraction state: 80% ethanol; 2) water extraction state: deionized water; 3) sodium chloride extraction state: 1 mol/L sodium chloride; 4) acetic acid extraction state: 2% acetic acid; 5) hydrochloric acid extraction state: 0.6 mol/L hydrochloric acid. Weigh 0.5 g of fresh leaves, add 20 ml of extraction solution for extraction, shake for 20 h at 250 rpm in a 30°C incubator, and centrifuge for 10 min at 3,500 rpm. Collect the supernatant into the centrifuge tube, repeat the above operation twice, and merge the extracts. The sediment is extracted with the next step, and the above steps are repeated. Finally, evaporate the five extracts to nearly dry and determine the content of Cd according to the method in *Determination of the Cadmium Content in Rice*.

### 2.5 Statistical analysis

One-way analysis of variance (ANOVA) test was applied to study the effect of foliar Si fertilizer on the growth, yield, and accumulation of Cd and Si of different rice varieties under Cd stress. The statistical and correlation analysis of the data was performed by SPSS19.0 and Excel 2019 software. The significance analysis was performed by Duncan’s test method, and figures were generated using Origin 2021.

## 3 Results

### 3.1 Effects of silicon on the rice growth and yield under cadmium stress

**Figure 1:**
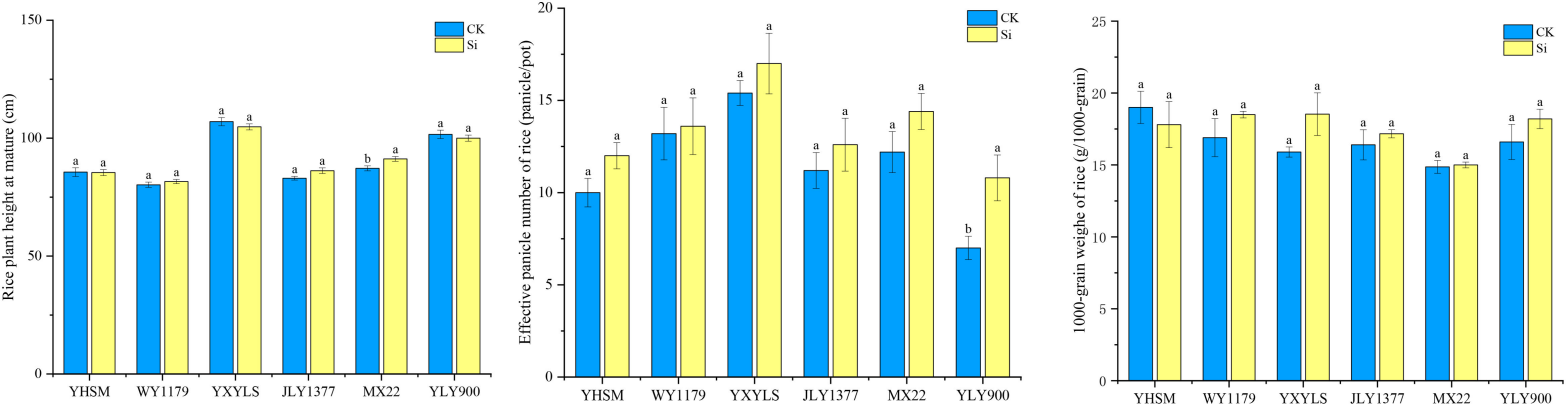
Effect of foliar spraying of silicon (Si) fertilizer on the effective number of panicle, 1,000-grain weight, and plant height of different rice varieties. The data are shown as mean ± standard error (n = 3). Different letters indicated that there were significant differences among treatments of the same rice variety (P < 0.05).

As shown in **Table 2**, under check (CK) conditions, the yields of different varieties from high to low are as follows: WY1179, JLY1377, YXYLS, YLY900, MXZ2, and YHSM. The yield of hybrid varieties is higher than that of conventional varieties. Among the four hybrid varieties, WY1179 obtained the highest yield, followed by JLY1377, YXYLS, and YLY900, and the remaining varieties reported low yield. Furthermore, there was no significant difference between the yield of rice varieties treated by Si spraying and the control, indicating that Si did not increase the yield of rice under the experimental conditions. **Figure 1** also shows that compared with CK, after applying Si, the effective panicle number of YLY900 increased by 54.3% significantly, and the other five varieties had no significant change. Except for MXZ2, the plant height was significantly increased by 4.6%, and the other varieties had no significant difference. In addition, the 1,000-grain weight of the six varieties was not significantly different from that of CK (P > 0.05).

**Table 2:** Response of foliar application of Si fertilizer on the grain yield (g pot^-1^) of different varieties of rice.

Rice Varieties	CK treatment	Si treatment
Yuehesimiao (YHSM)	4.81 ± 1.04a	6.50 ± 1.19a
Wuyou1179 (WY1179)	12.14 ± 3.98a	13.02 ± 0.13a
Yexiangyoulisi (YXYLS)	11.26 ± 2.37a	12.47 ± 1.24a
Jingliangyou1377 (JLY1377)	11.29 ± 1.36a	12.82 ± 1.63a
Meixiangzhan2 (MXZ2)	9.30 ± 0.41a	10.58 ± 2.94a
Yliangyou900 (YLY900)	10.16 ± 1.80a	10.61 ± 2.29a

The data are shown as mean ± standard error (n = 3). Different letters indicated that there were significant differences among treatments of the same rice variety (P < 0.05).

**Figure 2:**
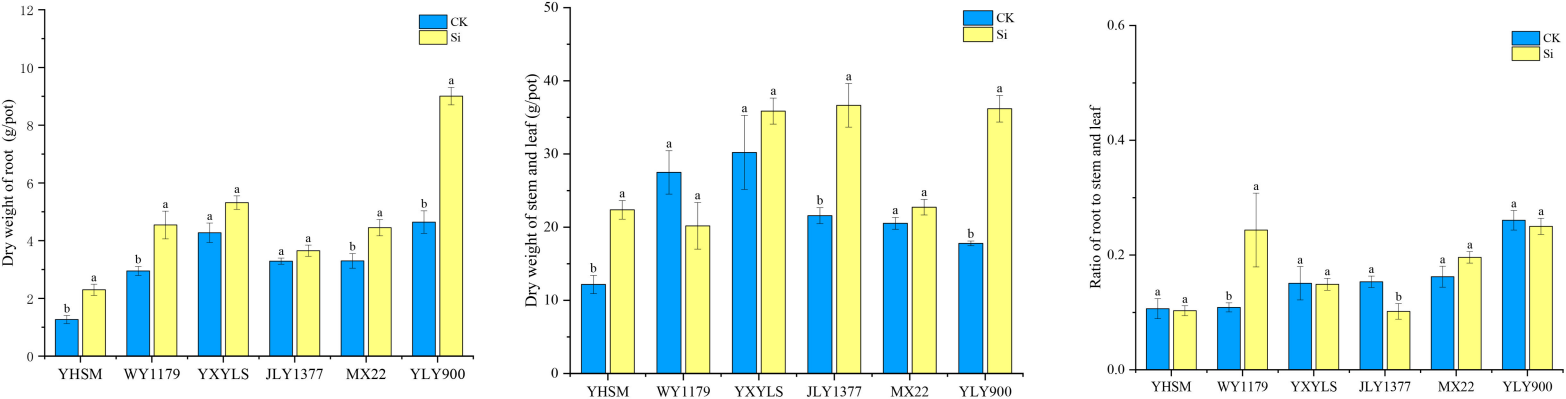
Response of foliar spraying of silicon (Si) fertilizer on the dry weight of the root, stem, and leaf and the ratio of the roots to the stems and the leaf of rice varieties at maturity stage. The data are shown as mean ± standard error (n = 3). Different letters indicated that there were significant differences among treatments of the same rice variety (P < 0.05).

Compared with CK, the root dry weight of YLY900, WY1179, MXZ2, and YHSM after Si spraying increased by 34.8%~94.0%, while the root dry weight of the other varieties did not change significantly (**Figure 2**). In addition, compared with CK, Si application showed that the dry weight of the stems and leaves of JLY1377, YLY900, and YHSM varieties increased significantly by 69.9%–103.6%. As for the root/shoot ratio, compared with CK, WY1179 increased significantly by 123.9% after Si spraying, while JYL1377 decreased significantly by 33.7%.

### 3.2 Cadmium content in different parts of the rice plant

Among the six varieties under CK conditions, WY1179 and YLY900 had the highest Cd content in brown rice, and JLY1377, MXZ2, YHSM, and YXYLS varieties had the lowest Cd content. After applying Si, the Cd content in brown rice of WY1179 and YHSM increased by 15.7% and 24.1%, respectively, while that of JLY1377, MXZ2, and YLY900 decreased by 34.3%–65.7%. Therefore, the response mechanisms of different varieties to Si are different. According to the difference in the trend of Cd content in brown rice after Si application, the six varieties were divided into two categories for analysis and comparison. The first category was negatively regulated by Si to inhibit the accumulation of Cd in brown rice, called Si-inhibited varieties, such as JLY1377, MXZ2, and YLY900. Under Si application conditions, the Cd content of brown rice in YXYLS showed a decreasing trend, so YXYLS was also classified as a Si-inhibited variety. The second category is positively regulated by Si and promotes Cd accumulation in brown rice, called Si-stimulated varieties, such as WY1179 and YHSM.

**Figure 3:**
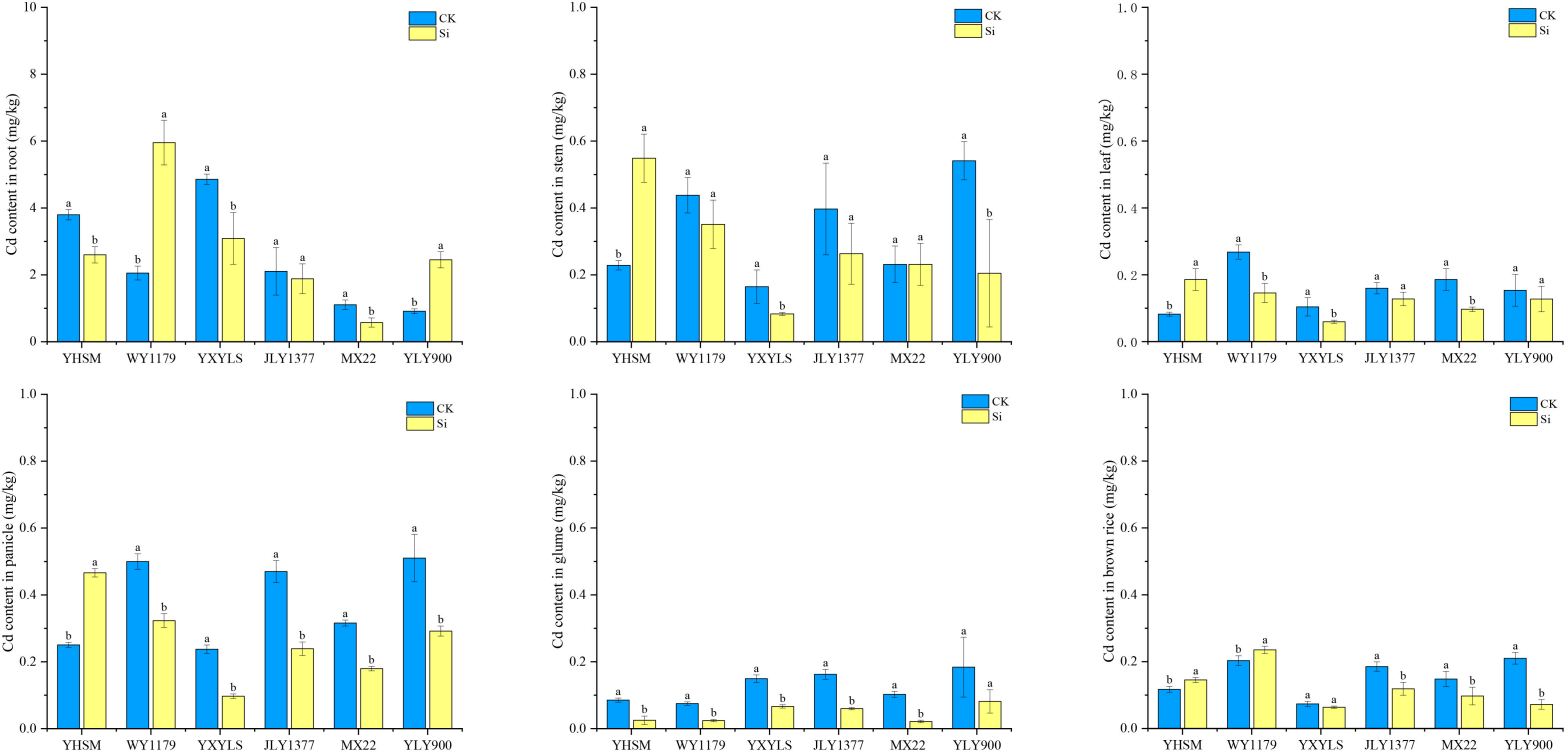
Effect of foliar spraying of silicon (Si) fertilizer on the cadmium (Cd) content in the root, stem, leaves, panicle, glumes, and brown rice of different rice varieties under Cd stress. The data are shown as mean ± standard error (n = 3). Different letters indicated that there were significant differences among treatments of the same rice variety (P < 0.05).

**Figure 3** showed that there are significant differences in Cd content in different parts of rice under the CK condition. The Cd content in the root is the highest, followed by that of the stem and panicle, and the Cd content in the leaf, glume, and brown rice is lowest. For Si-inhibited varieties, the Cd content in the roots, stems, leaves, panicles, and glumes of MXZ2 and YXYLS significantly decreased by 36.4%–79.2% after Si application, the Cd content in the stems and glumes of JLY1377 significantly decreased by 49.1% and 63.1%, respectively, and the Cd content in the stems and panicles of YLY900 significantly decreased by 62.2% and 42.8%, respectively. For Si-stimulated varieties, Si application significantly increased the Cd content of YHSM stems, leaves, and panicle stems by 86.2%~140.1% and the Cd content of WY1179 roots by 189.8%, with no significant change in the other parts.

The results of Cd transport coefficient analysis showed that Si spraying on leaves decreased the upward transport coefficient of Cd in Si-inhibited varieties. Among them, the decrease of the leaf-panicle transport coefficient of YXYLS and JLY1377, the stem-leaf and panicle-glume transport coefficients of MXZ2, and the root-stem and panicle-grain transport coefficients of YLY900 after foliar Si application was the main reason for inhibiting Cd accumulation in rice (**Table 3**). For Si-stimulated varieties, the change of mobility coefficient of each part after Si application is also different. YHSM only shows an increasing trend in the root-stem transport coefficient after Si spraying, but the stem-panicle, leaf-panicle, panicle-glume, and panicle-grain transport coefficients are decreased. Si spraying increased the panicle-glume and panicle-grain transport coefficients of WY1179 but decreased the root-stem and stem-leaf transport coefficients, indicating that YHSM mainly promoted the accumulation of Cd in brown rice by increasing the transport efficiency from the root to the shoot. WY1179 mainly promoted the accumulation of Cd in brown rice by strengthening the panicle-grain seed transport capacity.

**Table 3:** Effects of foliar application of Si fertilizer on translocation factor (TF) values for Cd in different tissues of the six rice cultivars.

Rice varieties	Treatment	TF_root-stem_	TF_stem-leaf_	TF_stem-panicle_	TF_leaf-panicle_	TF_panicle-glume_	TF_panicle-grain_
YHSM	CK	0.060*	0.360	1.097*	3.049	0.342*	0.467*
	Si	0.213*	0.338	0.858*	2.579	0.055 *	0.312*
WY1179	CK	0.214*	0.620	1.147*	1.878	0.151*	0.410*
	Si	0.059*	0.438	0.939*	2.315	0.077*	0.737*
YXYLS	CK	0.034	0.651	1.548	2.388*	0.633	0.444*
	Si	0.029	0.714	1.166	1.631*	0.690	0.662*
JLY1377	CK	0.188	0.446	1.298	2.942*	0.354	0.397*
	Si	0.161	0.524	0.984	1.877*	0.256	0.497*
MXZ2	CK	0.213*	0.821*	1.455	1.747	0.324*	0.473
	Si	0.411*	0.422*	0.837	2.056	0.120*	0.545
YLY900	CK	0.601*	0.280	0.932	3.385	0.338	0.428*
	Si	0.086*	1.094	2.990	2.470	0.271	0.245*

TF_root-stem_ = Cd_stem_/Cd_root_; TF_stem-leaf_ = Cd_leaf_/Cd_stem_; TF_stem-panicle_ = Cd_panicle_/Cd_stem_; TF_leaf-panicle_ = Cd_panicle_/Cd_leaf_; TF_panicle-glume_ = Cd_glume_/Cd_panicle_; TF_panicle-grain_ = Cd_grain_/Cd_panicle_. Values are means (n = 3). The * indicated that there were significant differences among treatments of the same rice variety (P < 0.05).

### 3.3 Silicon content in different parts of the rice plant

**Figure 4:**
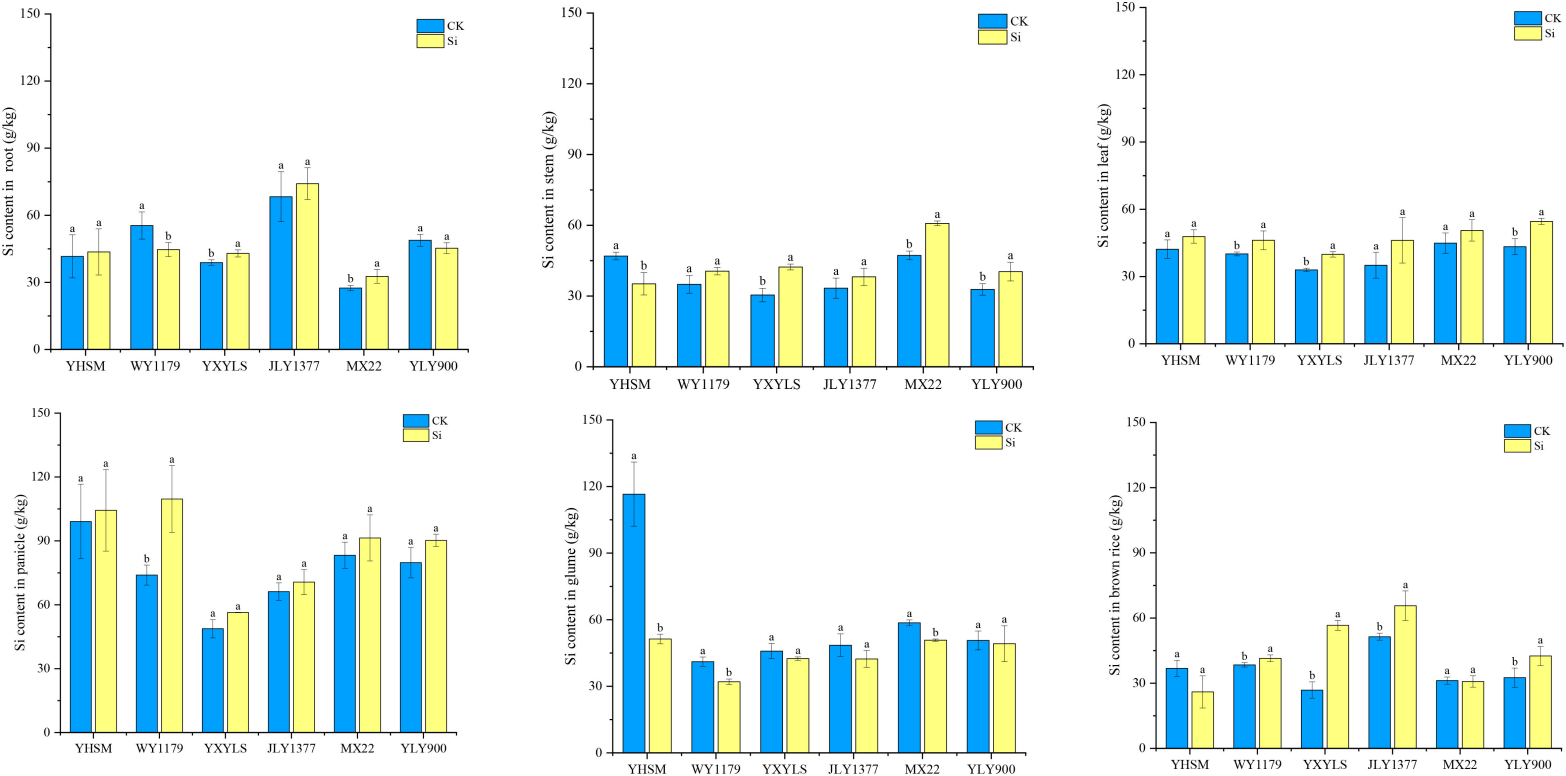
Effect of foliar spraying of silicon (Si) fertilizer on the Si content in the roots, stem, leaf, panicle, glumes, and brown rice of different rice varieties under cadmium (Cd) stress. The data are shown as mean ± standard error (n = 3). Different letters indicated that there were significant differences among treatments of the same rice variety (P < 0.05).

Under CK conditions, YHSM followed by YLY900 had the highest Si content in brown rice, and WY1179, JLY1377, MXZ2, and YXYLS varieties had the lowest Si content. For the Si-inhibited varieties, compared with CK, the Si content in the roots and stems of YXYLS and MXZ2 significantly increased by 10.6%–18.9% and 28.8%–39.1%, respectively, after Si application. The Si content in the stems of YLY900 also increased significantly (**Figure 4**). Compared with CK, the Si content of the glume shells showed a decreasing trend, and MXZ2 decreased significantly by 13.38%. Moreover, the Si content in brown rice in YXYLS, JLY1377, and YLY900 was significantly increased by 27.7%–111.2% after applying Si. For Si-stimulated varieties, the Si content of the roots of WY1179 decreased by 19.4% significantly after Si application compared with CK. The Si content of the stems of YHSM decreased by 25.1%. The Si content of the glumes of YHSM and WY1179 decreased by 55.97% and 22.11%, respectively.

### 3.4 Correlation results

The correlation analysis in **Table 4** shows that the Cd content in rice grains is positively correlated with that in the stems, leaves, and panicles (P < 0.05) but not significantly correlated with that in the glumes and roots. The Cd content in the panicles was significantly positively correlated with that in the stems (r^2^ = 0.860) and leaves (r^2^ = 0.774), indicating that Cd in brown rice mainly came from the panicles, and the Cd content in the panicles was positively regulated by that in the stems and leaves. The Si content in the stem was significantly negatively correlated with the Cd content in the panicles, glumes, and grains, indicating that Si in the stem inhibited Cd accumulation in the ears and above. Si inhibits the upward transport of Cd in the stems, which is of great significance to reduce the Cd content in brown rice.

**Table 4:** Correlation analysis of Cd and Si content in different parts of rice varieties.

	Si_Root_	Si_Stem_	Si_Leaf_	Si_Panicle_	Si_Glume_	Si_Rice_	Cd_Root_	Cd_Stem_	Cd_Leaf_	Cd_Panicle_	Cd_Glume_	Cd_Rice_
Si_Root_	1											
Si_Stem_	-0.356^*^	1.000										
Si_Leaf_	0.012	0.508^**^	1.000									
Si_Panicle_	-0.109	0.392^**^	0.615^**^	1.000								
Si_Glume_	-0.159	0.310^*^	0.062	0.295^*^	1.000							
Si_Rice_	0.688^**^	-0.017	0.046	-0.169	-0.158	1.000						
Cd_Root_	-0.032	-0.267	-0.222	0.095	0.007	0.004	1.000					
Cd_Stem_	0.369^**^	-0.255	0.226	0.390^**^	-0.041	-0.146	-0.163	1.000				
Cd_Leaf_	0.242	-0.217	0.146	0.213	-0.193	-0.090	-0.225	0.668^**^	1.000			
Cd_Panicle_	0.348^*^	-0.446^**^	0.019	0.230	-0.064	-0.196	-0.180	0.860^**^	0.774^**^	1.000		
Cd_Glume_	0.143	-0.425^**^	-0.392^**^	-0.424***	0.118	-0.002	-0.140	0.164	0.158	0.411^**^	1.000	
Cd_Rice_	0.258	-0.309^*^	-0.092	0.266	-0.172	-0.106	0.102	0.690^**^	0.608^**^	0.737^**^	0.211	1.000

Correlation is the comparison between different treatments of the same variety. *P < 0.05, **P < 0.01, ***P < 0.001.

### 3.5 Cadmium subcellular distribution in the leaf cell

**Figure 5:**
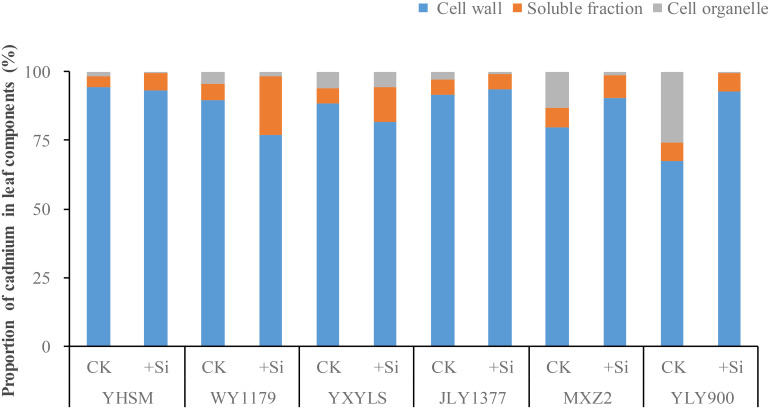
Effect of foliar spraying of silicon (Si) fertilizer on the proportion of cadmium (Cd) subcellular distributions in the leaf of different rice varieties.

It can be seen from **Figure 5** that leaf Cd mainly exists in the cell wall (77.07%–93.48%), followed by soluble components (3.87%–21.47%), and only a small part of Cd exists in the cell membrane (organs) and is the lowest (0.38%–17.09%). As for the Si-stimulating variety (WY1179), Si application decreased the distribution ratio of Cd in the cell wall and soluble components compared with CK, while Si-inhibiting varieties (MXZ2 and YLY900) showed the opposite trend by Si spraying.

### 3.6 Cadmium chemical form in rice leaves

**Figure 6:**
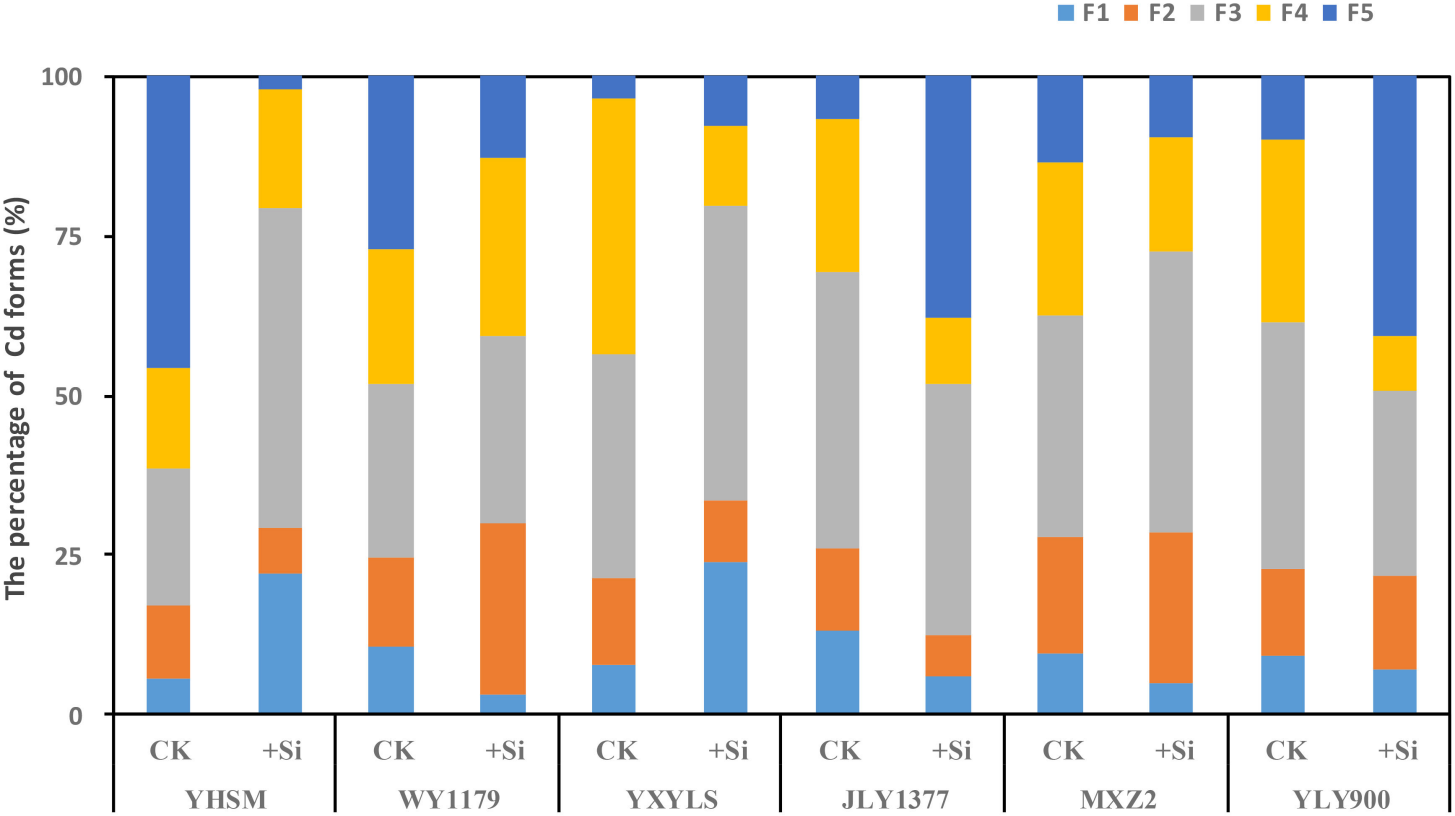
Effect of foliar spraying of Si fertilizer on the distribution proportion of cadmium (Cd) forms in the leaf of different rice varieties. F1, ethanol extraction state of Cd; F2, water extraction state of Cd; F3, sodium chloride extraction state of Cd; F4, acetic acid extraction state of Cd; and F5, hydraulic acid extraction state of Cd.

The different chemical forms of Cd in the leaves of the above six rice varieties can be extracted by different extracting agents. The proportions of different Cd chemical forms are shown in **Figure 6**. As for Si-stimulating varieties, Cd forms with strong mobility (such as small molecular soluble salt and small molecular organic binding state) increased significantly after spraying Si, while the proportion of Cd forms with weak mobility (such as phosphate-binding state and oxalate-binding state) decreased significantly. For example, after Si spraying, the ethanol-extracted Cd of YHSM was 2.98 times higher than that of CK, while the HCl-extracted Cd was only 4.5% of CK. The proportion of various Cd forms in the leaves of Si-inhibited varieties showed opposite trends after Si spraying. For example, after spraying Si, the ethanol-extracted Cd of JLY1377 decreased by 54.77% compared with CK, and the HCl-extracted Cd increased by 473.8% compared with CK.

## 4 Discussion

### 4.1 Effect of silicon on yielding traits of rice under cadmium stress

Cd can inhibit the growth of crops by disrupting the normal physiological and molecular mechanisms of crops (Rizwan et al., 2016), and Si spraying on the leaf surface will alleviate this restriction to some extent. In this study, foliar Si spraying promoted rice growth under Cd stress. Compared with CK, foliar Si spraying generally increased the dry weight of the roots, stems, and leaves of rice, with a higher increase in YLY900, and also increased the plant height of MXZ2 rice. Jiang and Zhang (2002) found that the increase of photosynthetic capacity and dry matter productivity of rice after the application of Si fertilizer may be the reason for the increase of dry matter weight of rice leaves, stem sheath, and panicle. Li et al. (2018) showed that exogenous Si application could reduce the inhibition of Cd stress on rice growth by promoting photosynthesis and antioxidant enzyme activity.

Si has a certain effect of promoting rice yield increase, but it varies with various factors (Huang et al., 2013). In this study, the results showed that the 1,000-grain weight of rice had no significant change after spraying Si fertilizer on the leaf surface, but the number of effective panicles and rice yield showed a certain increasing trend from the perspective of yield components. Wang et al. (2007) found that the effect of Si application on yield increase was mainly realized by the increase of effective panicles and grains per panicle, while 1,000-grain weight had little effect. It has also been found that Si fertilizer can improve rice yield by increasing the number of tillers, total spikelets, and seed setting grains of rice and increasing the percentage of panicles (Yang et al., 2021; Shang et al., 2009). Therefore, we speculate that the effect of spraying Si fertilizer on leaves on the number of effective panicles may be an important reason for the effect of Si fertilizer on rice yield in this experiment.

### 4.2 Foliar silicon spraying reduced cadmium accumulation in silicon-inhibited rice

Foliar spraying with Si slows down the toxic effects of Cd by fixing Cd in the leaves and stems. Shi et al. (2005) and Zhang et al. (2008) found that the accumulation of Si in the root can reduce the transportation of apoplast, provide metal-chelating points, and reduce the content of different forms of Cd in the apoplast, especially free Cd, thus reducing the absorption of Cd and the transfer from the root to the upper ground. It has also been pointed out that Si may affect the redistribution of Cd after it enters the rice body and inhibit the toxicity of Cd by reducing the upward transport of Cd (Wang X. M. et al., 2016). This study also found that the root-stem, stem-leaf, and leaf-panicle transport of Si-inhibited varieties was inhibited to varying degrees after Si spraying, resulting in a significant decrease in Cd content in rice panicles. Through further correlation analyses, it was found that Cd in brown rice mainly came from the ears, while Si in the stems inhibited the accumulation of Cd in the ears. Zhang et al. (2016) also found that under Cd stress, Si application significantly reduced the Cd transfer coefficient and enrichment coefficient and reduced the Cd content of brown rice. Therefore, for Si-inhibited varieties, the accumulation of Si in roots and stems increased after Si spraying, which inhibited the upward transport of Cd, thereby reducing the accumulation of Cd in rice.

The significant difference in the absorption and accumulation of Cd in different rice varieties, which is related to the subcellular and chemical form distribution of Cd, is different in rice (Li et al. 2003; Ma et al., 2015). Gu et al. (2011) believe that the highest content of Cd in the cell wall is due to the fact that the cell wall contains a large number of negatively charged groups. These groups are precipitated and complexed with positively charged heavy metal ions, so that most of the Cd is bound in the cell wall. Rice can alleviate the toxic effects of Cd by combining Cd with Si in the cell wall and changing the redox potential (Shao et al., 2017; Chen et al., 2019). Combining with the negatively charged hemicellulose form of Si can inhibit the uptake of Cd by rice cells (Zhou and Wang, 1999). The effect of Si on different migration states of Cd in rice also shows that the formation of Cd is hard to migrate and thus reduced the migration of Cd in rice (Peng et al., 2017). After foliar spraying of Si fertilizer in Si-inhibited varieties, the Cd content in the organelles decreased, and the Cd content in the cell wall increased significantly. At the same time, the ratio of the ethanol-extracted state and deionized water-extracted state with strong flow activity decreased, while the proportion of weak flow active extraction state (acetic acid-extracted state and hydrochloric acid-extracted state) increased. Therefore, for Si-inhibited cultivars, the reason for the reduction of grain Cd content after Si application may be that it reduces the Cd content in organelles, increases the adsorption of Cd on the cell wall, and promotes the refractory migration of acetic acid-extracted and hydrochloric acid-extracted Cd forms and reduced bottom-up transport of Cd in the rice, thereby reducing Cd transport to the grain and ultimately reducing Cd content in brown rice.

### 4.3 Foliar silicon spraying promoted cadmium accumulation in silicon-stimulated rice

Si spraying on the leaves significantly increased the Cd content in brown rice of WY1179 and YHSM. We call these rice varieties Si-stimulated. The Cd in brown rice comes from two main sources: firstly, the translocation of Cd absorbed by the roots into brown rice through xylem transport and, secondly, the reactivation of Cd accumulated in the leaves, especially the sword leaves (Tamai and Ma, 2008). Our results showed that the Si content in the stem of YHSM and the root of WY1179 decreased rather than increased after leaf spraying. At the same time, the Cd content in the stems of YHSM and the roots of WY1179 was significantly increased, and the transport coefficients of the rhizomes and panicle-grain were increased, which might promote the accumulation of Cd in rice. In addition, after Si application, the Cd content in the glume of WY1179 and YHSM decreased, indicating that Si application may reduce the distribution proportion of Cd in Si-stimulated varieties’ glume, thus promoting the accumulation of Cd in rice. Our results also showed that the content of Cd in the cell wall of the Si-stimulated variety (WY1179) was significantly reduced, while the proportion of Cd in the soluble fraction was increased. In addition, the extracted Cd (acetic acid and hydrochloric acid extracts) with weak flow activity became ethanol extracts and deionized water extracts with a strong flow activity. In this study, the higher content and distribution of Cd in the ethanol-extracted and NaCl-extracted states in the leaves of the Si-stimulated variety YHSM allowed more active Cd to be transported to the grain during the filling period, which may have contributed to its higher grain Cd content. Differences among varieties are also small in the roots, stems, and leaves but larger in the grains (Liu et al., 2007). Therefore, for Si-stimulated varieties, the increase in the Cd content of brown rice after Si application may be due to the increased Cd content in the cell organelles, reduced Cd adsorption on the cell wall and promotion of the production of the mobile active ethanol-extracted and deionized water-extracted states of hard, and increased Cd uptake by the rice root system and bottom-up transport of Cd in rice, thus facilitating Cd translocation to the grain and ultimately increasing the Cd content of brown rice.

## 5 Conclusions

In this study, we compared the effects of foliar spraying of Si fertilizer on the absorption, transport, and accumulation of Cd and Si as well as the yield of six different rice varieties, so as to further explore the response mechanism of different rice varieties to Si. The results showed that foliar spraying of Si fertilizer generally increased the dry weight of the root, stem, and leaf and promoted the growth of rice, but the effective panicle number and yield of rice had no significant change, which may be related to the low Si concentration. However, the response of Cd accumulation to Si in different rice varieties was significantly different. The content of Cd in the grain of Si-stimulated rice was increased by Si, while that of Si-inhibited rice was inhibited by Si. We have preliminarily clarified the mechanism of response difference. For Si-inhibited varieties, after applying Si, the content of Cd in the leaf cell wall and the chemical binding state with weak mobility are increased, the mobility and activity of Cd are reduced, and the bottom-up transport of Cd in rice straw is reduced; finally, the content of Cd in brown rice is reduced. For Si-stimulated varieties, Si application increased the content of Cd in leaf organelles and the chemical binding state with strong mobility and increased the uptake of Cd by rice roots and the transport of Cd from bottom to top in rice straw, thus promoting the transport of Cd to grains. Therefore, we suggest that future large-scale spraying of Si fertilizer on Cd-contaminated farmlands should be combined with rice varieties in order to achieve the desired effect.

## Data availability statement

The raw data supporting the conclusions of this article will be made available by the authors, without undue reservation.

## Author contributions

JZ and BY performed experiment and data curation, XW and LC done formal analysis and software, KA - writing review and editing, ST - Methodology, HL and JH - software, RW - writing review and editing, BH - supervision, and funding acquisition. All authors contributed to the article and approved the submitted version.
